# Single-channel EEG signal extraction based on DWT, CEEMDAN, and ICA method

**DOI:** 10.3389/fnhum.2022.1010760

**Published:** 2022-09-21

**Authors:** Qinghui Hu, Mingxin Li, Yunde Li

**Affiliations:** ^1^School of Computer Science and Engineering, Guilin University of Aerospace Technology, Guilin, China; ^2^School of Computer Science and Information Security, Guilin University of Electronic Technology, Guilin, China; ^3^School of Electronic Information and Automation, Guilin University of Aerospace Technology, Guilin, China

**Keywords:** electroencephalogram, discrete wavelet transform, empirical mode decomposition, independent component analysis, sample entropy

## Abstract

In special application scenarios, such as portable anesthesia depth monitoring, portable emotional state recognition and portable sleep monitoring, electroencephalogram (EEG) signal acquisition equipment is required to be convenient and easy to use. It is difficult to remove electrooculogram (EOG) artifacts when the number of EEG acquisition channels is small, especially when the number of observed signals is less than that of the source signals, and the overcomplete problem will arise. The independent component analysis (ICA) algorithm commonly used for artifact removal requires the number of basis vectors to be smaller than the dimension of the input data due to a set of standard orthonormal bases learned during the convergence process, so it cannot be used to solve the overcomplete problem. The empirical mode decomposition method decomposes the signal into several independent intrinsic mode functions so that the number of observed signals is more than that of the source signals, solving the overcomplete problem. However, when using this method to solve overcompleteness, the modal aliasing problem will arise, which is caused by abnormal events such as sharp signals, impulse interference, and noise. Aiming at the above problems, we propose a novel EEG artifact removal method based on discrete wavelet transform, complete empirical mode decomposition for adaptive noise (CEEMDAN) and ICA in this paper. First, the input signals are transformed by discrete wavelet (DWT), and then CEEMDAN is used to solve the overcomplete and mode aliasing problems, meeting the a priori conditions of the ICA algorithm. Finally, the components belonging to EOG artifacts are removed according to the sample entropy value of each independent component. Experiments show that this method can effectively remove EOG artifacts while solving the overcomplete and modal aliasing problems.

## Introduction

EEG (Saeidi et al., 2021) is a non-linear and non-stationary electrophysiological signal of the central nervous system that contains rich information about brain activity. It is an important information source for human brain research, disease diagnosis and rehabilitation engineering. It also has rich rhythmic activity and can be used to characterize the dynamic changes in brain function. EEG signal acquisition equipment is required to be more portable and easier to use with the application of brain computer interfaces (BCIs). The fewer the number of channels is, the better in some special application scenarios, such as portable anesthesia depth monitoring, portable emotional state recognition (Wan et al., 2022) and portable sleep monitoring (Kwon et al., 2021), in which only a single channel is needed. However, EEG signals are easily contaminated by physiological and non-physiological artifacts, such as EOG (Miao et al., 2021), electromyogram (EMG) (Meng et al., 2022) and electrocardiogram (ECG) artifacts (Mourad, 2022). As a result, the brain function information in EEG signals is concealed by artifacts, which results in inaccurate classification. EOG artifacts have the highest amplitude and strongest randomness compared with the other two kinds of artifacts. The presence of artifacts makes EEG signals prone to obvious distortion (Jiang et al., 2019; Gu et al., 2021), interferes with the inherent information expression of neuronal electrical activity, weakens the signal-to-noise ratio and increases the difficulty of preprocessing in the recording process (Dora and Biswal, 2020; Liu et al., 2021; Sun et al., 2021).

In recent years, many classical methods, such as average artifact regression analysis (Semlitsch et al., 1986), principal component analysis (PCA) (Vigon et al., 2000), and ICA (Makeig et al., 1996; Jiang et al., 2020; Yuan et al., 2020), have been widely used to remove EOG artifacts from multichannel EEG signals. In the first method, it is assumed that the conductivity between the electrode collecting EOG signal and other electrodes remains unchanged. Then, the correlation between the EOG channel and other channels is estimated, and the EOG signals are removed from each channel according to the conductivity. In the PCA method, the EEG and EOG signals need to be recorded at the same time during the experiment, and the artifacts are removed by analyzing the main components. Different from the first method, the ICA method obtains each independent source signal and separates the features only according to its statistical characteristics and the observed signal when the source signal and transmission channel parameters are unknown (Li et al., 2013).

The above methods are all difficult to apply to a single-channel portable BCI system, as they require more EEG channels to achieve a better separation effect. Kumar proposed a method that removes single-channel EOG artifacts by using a wavelet transform soft threshold (Kumar et al., 2008). However, this method requires much prior knowledge and is highly subjective, and directly removing the wavelet coefficients will negatively impact the components of the EEG source signal. Mammone proposed a WT-ICA algorithm to remove EOG artifacts based on both wavelet transform (WT) and ICA (Mammone et al., 2012). This algorithm meets the ICA a priori condition by wavelet decomposition of the single-channel EEG signal. However, the wavelet transform not only increases the observation signals but also decomposes the source signal into several subsource signals, which leads to the overcomplete problem. The empirical mode decomposition (EMD) (Wu and Huang, 2011) proposed by Huang is an adaptive time-frequency decomposition method that can better deal with non-linear and non-stationary signals (Park et al., 2011). However, there will be intermittent phenomena due to abnormal events (such as tip signals, pulse interference and noise) in the EMD process, which results in mode aliasing and causes the IMF component to lose its specific physical significance.

Aiming at the problem that the ICA method cannot be used to solve the overcomplete and modal aliasing problems caused by using empirical mode decomposition to make the number of observed signals greater than that of the source signals in the process of single-channel EOG artifact removal, we propose a single-channel EOG artifact removal algorithm (DWT-CEEMDAN-ICA) based on DWT, CEEMDAN and ICA in this paper. First, the source signals are transformed by a discrete wavelet and decomposed by the CEEMDAN method so that they meet the a priori condition of the ICA algorithm, which solves the overcomplete and mode aliasing problems. Experiments show the effectiveness and stability of this algorithm compared with the other EOG artifact removal algorithms.

This paper is organized as follows. Related work is introduced in Section 2. In Section DWT-CEEMDAN-ICA algorithm, the proposed method is described in detail. Experiments are presented in Section 4, and some concluding remarks are given in the last section.

## Related works

### Discrete wavelet transform

The idea of wavelet transform is to gradually refine the signal at multiple scales through expansion and translation operations so that it can be subdivided according to time in the high-frequency domain and subdivided according to frequency in the low-frequency domain. The EEG signal after wavelet transform has better frequency resolution corresponding to the two different domains, which automatically meets the requirements of time-frequency signal analysis and focuses on any detail. The window function is given by:


(1) $$\begin{array}{l} \psi_{a,b}\left(t\right)=\frac{1}{\sqrt{a}}\psi \left(\frac{t-b}{a}\right) \end{array}$$

Where *a* and *b* are the scale displacement and time displacement, respectively.

In the DWT method, the parameter α, τ of wavelet basis function ψ(α, τ) needs to be limited to discrete points. The basis function is:


(2) $$\begin{array}{l} \psi_{j,k}\left(t\right)=2^{-\frac{j}{2}}\psi \left(2^{-j}t-k\tau_{0}\right) \end{array}$$

Where *j* and *k* are the frequency resolution and time translation, respectively; then, the DWT at this time is:


(3) $$W_{\psi }f(j,k)=\int^{\;}f(t)\psi_{j,k}^{*}(t)dt$$

### Complete empirical mode decomposition

The idea of Empirical mode decomposition (EMD) is to decompose the signal step by step according to the fluctuation or trend to produce a series of data sequences with different characteristic scales. Each sequence is called an intrinsic mode function (IMF) (Boda et al., 2021) and meets the following conditions: The number of extreme points and zero crossings in the whole data segment is equal or has no more than one difference. The average value of the upper and lower packet routes formed by the local maximum and minimum points is zero and locally symmetrical about the time axis at any time. However, due to the large amount of noise, jumping change of the time scale and boundary effect in the actual signal, the phenomenon of mode aliasing will be caused in the process of EMD. The EMD formula is as follows:


(4) $$\begin{array}{l} x\left(t\right)=\sum_{i=1}^{N}a_{n}\left(t\right)+r_{n}\left(t\right) \end{array}$$

Where *a*_*n*_(*t*) is the nth-order IMF, *r*_*n*_(*t*) is the remainder, and *N* is the number of IMFs.

To solve the problem of mode aliasing in the EMD method, Torres et al. proposed a complete empirical mode decomposition (CEEMDAN) algorithm that can adapt to noise (Xu et al., 2018). White noise is added to the residual value, and the mean value is calculated for each IMF component and then iterated step by step.

The method is described as follows:

EEG signals and EOG artifacts partially coincide in the time domain and frequency domain of the low-frequency band. If the artifacts are directly removed from the result of the discrete wavelet transform, part of the EEG signal will be lost, resulting in distortion. In addition, the a priori condition of the ICA method will no longer be met. Therefore, we use the Algorithm 1 (CEEMDAN method) to decompose the wavelet coefficients after wavelet transform into several IMFs.

**Algorithm 1:** CEEMDAN

1:	Add Gaussian white noise to the original signal: (5) $$\begin{array}{l} x_{j}\left(t\right)=x\left(t\right)+\sigma_{0}w^{j}\left(t\right) \end{array}$$ Where σ_0_ is the standard deviation of the noise. *w*^*j*^(*t*) is the white noise added by the j-th decomposition, which is subject to the *N*(0, 1) distribution.
2:	*x*_*j*_(*t*) is decomposed by EMD *N* times. After the first decomposition, the mean value is taken to obtain the first-stage modal component $\tilde{IMF_{1}}\left(t\right)$, as shown in Equation (6): (6) $$\begin{array}{l} \tilde{IMF_{1}}\left(t\right)=\frac{1}{N}\sum_{j=1}^{N}IMF_{1}^{j}\left(t\right)=\tilde{IMF_{1}}\left(t\right) \end{array}$$
3:	Obtain the first-stage residual by Formula (7) (7) $$\begin{array}{l} r_{1}\left(t\right)=x\left(t\right)-\tilde{IMF_{1}}\left(t\right) \end{array}$$
4:	When the number of extreme points of *r*_1_(*t*) exceeds two, the first-stage residual *r*_1_(*t*) is added to the first-stage modal operator to form a new residual signal $r_{1}\left(t\right)+\sigma_{1}M_{1}\left[w^{j}\left(t\right)\right]$, and then EMD is carried out to obtain the second-stage modal component $\tilde{IMF_{2}}\left(t\right)$: (8) $$\begin{array}{l} \tilde{IMF_{2}}\left(t\right)=\frac{1}{N}\sum_{j=1}^{N}M_{1}\left\{r_{1}\left(t\right)+\sigma_{1}M_{1}\left[w^{j}\left(t\right)\right]\right\} \end{array}$$ Where σ_1_ represents the second-stage standard deviation of the noise and *M*_*a*_[·] is the stage IMF mode operator after EMD of the signal.
5:	Repeat step 4 until the residual can no longer be separated, and the original signal *x*(*t*) is decomposed as shown in Equation (9): (9) $$\begin{array}{l} x\left(t\right)=R\left(t\right)+\sum_{k=1}^{K}\tilde{IMF_{k}} \end{array}$$ where *K* and *k* represent the number of times and layers of modal decomposition, respectively. The k-stage residual *r*_*k*_(*t*) in the kth layer decomposition is calculated by Equation (10): (10) $$\begin{array}{l} r_{k}\left(t\right)=r_{k-1}-\tilde{IMF_{k}}\left(t\right) \end{array}$$ The *k*+1 stage modal component is calculated by Equation (11): (11) $$\begin{array}{l} \tilde{IMF_{k+1}}\left(t\right)=\frac{1}{N}\sum_{j=1}^{N}M_{1}\left\{r_{k}\left(t\right)+\sigma_{k}M_{k}\left[w^{j}\left(t\right)\right]\right\} \end{array}$$

### Independent component analysis and sample entropy

The original signal collected through the electrode is the linear instantaneous mix of EEG signals and EOG artifacts, which are independent of each other. Therefore, the ICA method can be used to decompose the original signal into multiple independent component spaces to realize the separation of EEG signals and EOG artifacts. Let *S* = [*S*_1_, *S*_2_, …, *S*_*M*_] be the *M* mutually statistically independent source signal. *X* = [*x*_1_, *x*_2_, …, *x*_*N*_] is the n-dimensional observation signal generated by the linear mixing of S through an unknown matrix A, i.e., *X* = *A* × *S*. Under the condition that both *A* and *S* are unknown, the ICA method uses the assumption that *X* and *S* are statistically independent to find a linear transformation separation matrix *W* to make the output signal approach *S* as much as possible. The FastICA (Chen et al., 2018) algorithm takes the maximum negative entropy as the search direction and can sequentially extract independent sources. In addition, it adopts fixed-point iteration to make the convergence faster and more robust. Therefore, we use this method to process the IMF and obtain the source signal *S*.

The EEG signal comes from brain bioelectric activity and contains much physiological and pathological information, while the EOG signal only represents eye movement and blinking. Compared with EOG signals, EEG signals have more complex characteristics. The higher the complexity is, the higher the corresponding entropy. Therefore, the components with high entropy can be extracted as EEG signals. Compared with the approximate entropy (Pincus and Goldberger, 1994; Li et al., 2010), the sample entropy has a better estimation effect in the time-domain statistics aspect (e.g., mean and variance) and can be used to calculate the mixed signal composed of the determined signal and random signal. Therefore, we use Algorithm 2 (Calculate sample-entropy) to remove the EOG artifacts in this paper. The Algorithm 2 is as follows:

**Algorithm 2:** Calculate sample-entropy *sampEn*(*m, r, N*)

1:	Reconstruct the m-dimensional space vector from the phase space (12) $$\begin{array}{l} X_{m}\left(i\right)=\left[x\left(i\right),x\left(i+1\right),\ldots ,x\left(i+m-1\right)\right]\\ S.t.\;1\leq i\leq N-m+1 \end{array}$$ Where *X*_*m*_(*i*) represents the phase space position of the ith point.
2:	Calculate the distance *d*[*X*_*m*_(*i*), *X*_*m*_(*j*)] between *X*_*m*_(*i*) and *X*_*m*_(*j*) (13) $$\begin{array}{l} d\left[X_{m}\left(i\right),X_{m}\left(j\right)\right]=\max_{k=0,1,\ldots ,m-1}\left(\left|x\left(i+k\right)-x\left(j+k\right)\right|\right)\;\\ S.t.\;k=0,1,\ldots ,m-1,i\neq j \end{array}$$
3:	For a given *X*_*m*_(*i*), count the number *B*_*i*_ of *X*_*m*_(*j*) satisfying *d*[*X*_*m*_(*i*), *X*_*m*_(*j*)] ≤ *r*, and then (14) $$\begin{array}{l} B_{i}^{m}\left(r\right)=B_{i}/\left(N-m\right)\\ S.t.\;1\leq i\leq N-m \end{array}$$
4:	Calculate the average of $B_{i}^{m}$ (15) $$\begin{array}{l} B^{m}=\sum_{i=1}^{N-m}B_{i}^{m}/\left(N-m+1\right) \end{array}$$
5:	Increase the dimension to *m* + 1. When the dimension is a finite value, calculate the sample entropy: (16) $$\begin{array}{l} S=-ln\left[B^{m+1}\left(r\right)/B^{m}\left(r\right)\right] \end{array}$$ Where *r* = (0.10 − 0.25)*D*, *D* is the standard deviation of {*x*(*i*)}.

## DWT-CEEMDAN-ICA algorithm

The innovation of this algorithm is that it can effectively solve the overcomplete and modal aliasing problem existing in the current single-channel EEG removal algorithm, and improve the retention of effective EEG information. The algorithm proposed in this paper is described in detail as follows:

1: The collected EEG signal is transformed by *db4* wavelet to obtain a low-frequency approximate component *A*_7_and seven high-frequency detail components *D*_*i*_(*i* = 1, …, 7) corresponding to different layers decomposed by 7 layers.
2: These components are single-branch reconstructed and CEEMDAN decomposed to obtain several IMFs, and FastICA decomposition is carried out to calculate several independent components and their sample entropy.
3: The component corresponding to the sample entropy satisfying the threshold discriminant proposed by Gomez-Herrero et al. (2006) is regarded as an EOG artifact and set to zero. Then, the inverse ICA transform is carried out to obtain the EEG signal after the artifact is removed. The threshold discriminant proposed by Gomez Herrero is as follows:
(17) $$\begin{array}{l} \varphi \left(k+1\right)-\varphi \left(k\right)<\varphi \left(k\right)-\varphi \left(k-1\right)\\ S.t.\;1<k\;\leq \;\left[N/2\right] \end{array}$$
  where φ(*k*) represents the entropy value of the *k*-th independent component sorted in ascending order. The ICA components corresponding to the top *k* entropy values are regarded as EOG artifacts where *k* takes the smallest integer which satisfies the above conditions.
4: Repeat steps 2–3 for the remaining wavelet coefficients to obtain all the signals after removing artifacts and then carry out wavelet reconstruction to obtain a complete EEG signal without artifacts.

**Figure 1:**
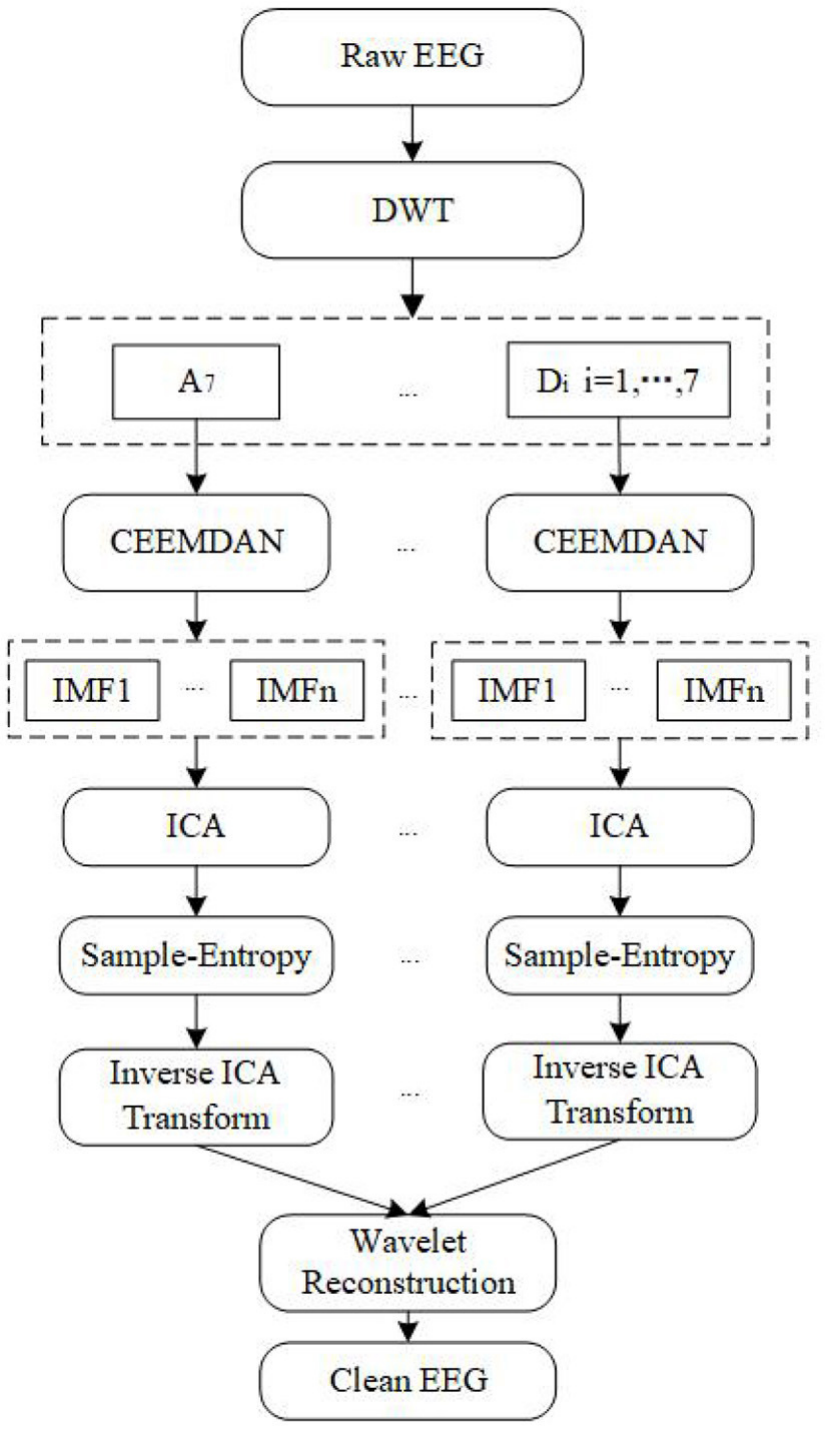
Flowchart of DWT-CEEMDAN-ICA.

Figure 1 shows the flowchart of the proposed algorithm.

## Experiments

In this section, a series of experiments are conducted to evaluate the efficiency of the proposed algorithm compared with a number of state-of-the-art algorithms used to remove EOG artifacts. The algorithms in our comparisons include the following:

1) Wave transform (WT) algorithm. First, the original signals are decomposed by wavelet transform, then the wavelet coefficients are denoised by the soft threshold method, and finally the signal is reconstructed by wavelet reconstruction.
2) WT-ICA algorithm. First, the original signals are decomposed by wavelet transform, then all wavelet coefficients are decomposed into independent components by the ICA algorithm, and finally the signals are reconstructed by ICA inverse transform of these components.
3) EMD-ICA algorithm. The original signals are first decomposed by empirical mode decomposition to acquire a series of IMFs, and then they are decomposed into independent components. Finally, the signals are reconstructed by ICA inverse transformation of these components.

The original signals used in the experiments are collected from the FP_1_ channel by a single electrode EEG machine (Mindwave) of NeuroSky Technology Company (NeuroSky). The device runs TGAM EEG module and adopts Bluetooth plus BLE dual-mode transmission. The sampling rate is 512 Hz, and the sampling time is 2 min. First, the ears and forehead of the subject were treated to remove grease and cutin to reduce the components of EMG artifacts. Then, the subject was allowed to close his eyes and rest for 2 min. During the collection, the subject was instructed to remain calm and blink several times in the natural manner. As a result, the collected signals contain fewer other artifacts, which can be approximately considered to contain only EEG signals and EOG artifacts.

The root mean square error *RMSE* and correlation coefficient R are introduced to evaluate the performance of the algorithms. The smaller the *RMSE* is, the closer the signal is to the original signals after artifact removal. The better the removal effect is, the larger the correlation coefficient *R* and the more complete the effective information of the signals that is retained.


(18) $$\begin{array}{l} RMSE=\sqrt{\frac{1}{n}\sum_{i=1}^{n}{\left[x_{i}-y_{i}\right]}^{2}} \end{array}$$

(19) $$\begin{array}{l} R=\frac{\sum_{i=1}^{n}\left(x_{i}-\bar{x}\right)\left(y_{i}-\bar{y}\right)}{\sqrt{\sum_{i=1}^{n}{\left(x_{i}-\bar{x}\right)}^{2}}\sqrt{\sum_{i=1}^{n}{\left(y_{i}-\bar{y}\right)}^{2}}} \end{array}$$

### Preprocessing

**Figure 2:**
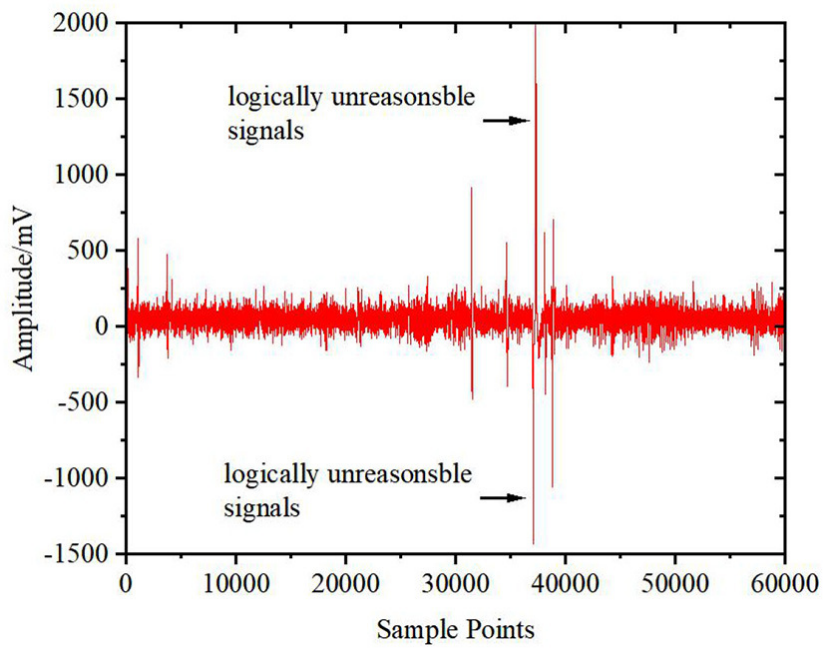
Original signal.

**Figure 3:**
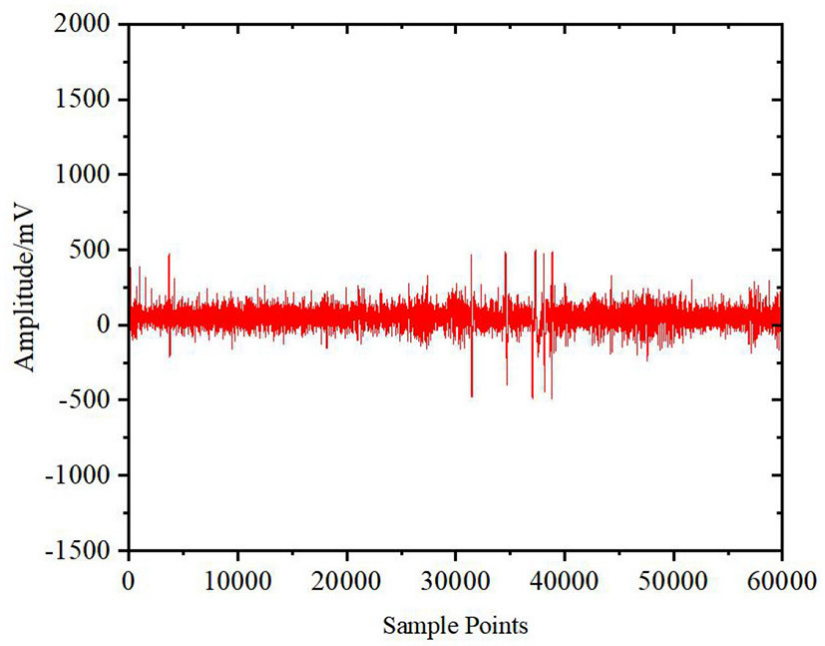
Signal after consistency check and interpolation.

Signal jump and mechanical noise are generated in the collection process, resulting in a certain amount of signals that are logically unreasonable and exceed the normal range of the original EEG signals. These signals affect the analysis quality of EEG signal data. Therefore, it is necessary to check the consistency and remove the unreasonable information from the original signals and estimate and supplement the defective signal by using the mean difference method of surrounding signals to restore continuity and time-frequency characteristics. The original signals and the signals after the consistency check and interpolation are shown in Figures 2, 3, respectively.

**Figure 4:**
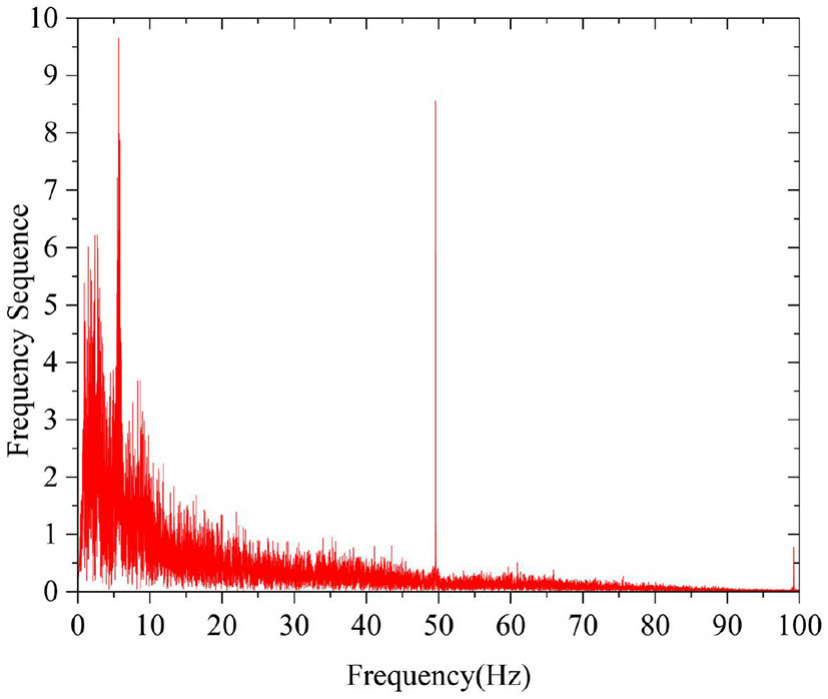
Spectrogram after bandpass filtering.

**Figure 5:**
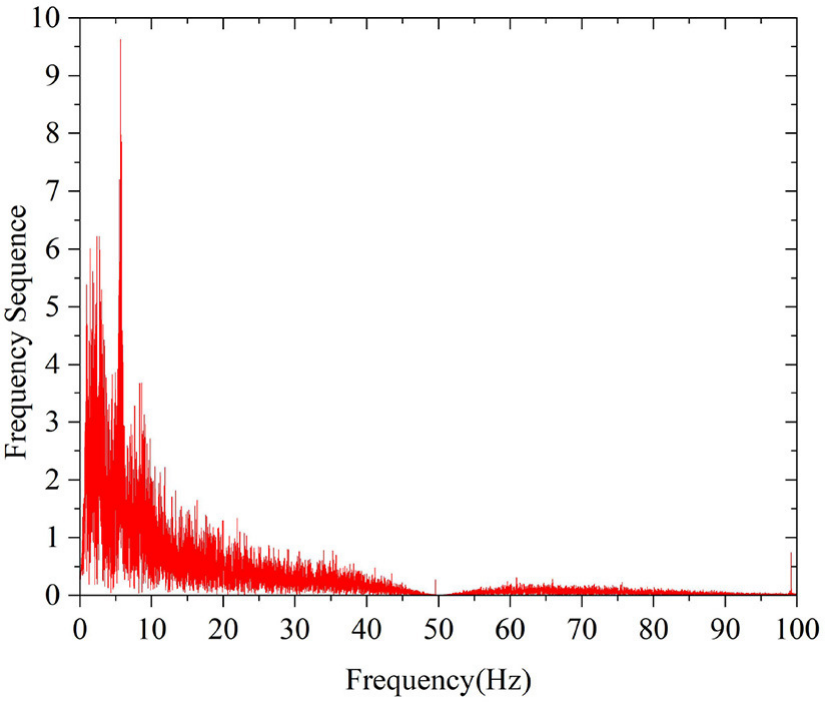
Spectrogram after bandstop filtering.

The frequency used to analyze the characteristics of EEG signals is mainly below 64 Hz, so it is necessary to filter and eliminate frequency interference by using a 0.05–64 Hz bandpass filter and a 50 Hz band-stop filter, respectively. Figures 4, 5 are the spectrum diagrams after bandpass and band-stop filtering, respectively.

### Experimental results and analysis

It can be seen from the preprocessed signals that the artifacts are mainly concentrated on the sampling points in the range of 30,000–40,000. Therefore, the points in this range were used to compare the effectiveness of removing EOG artifacts. The retention degree for effective information is obtained by comparing the correlation coefficients of the sampling points in the range of 50,000–55,000 before and after being processed by the DWT-CEEMDAN-ICA algorithm.

**Figure 6:**
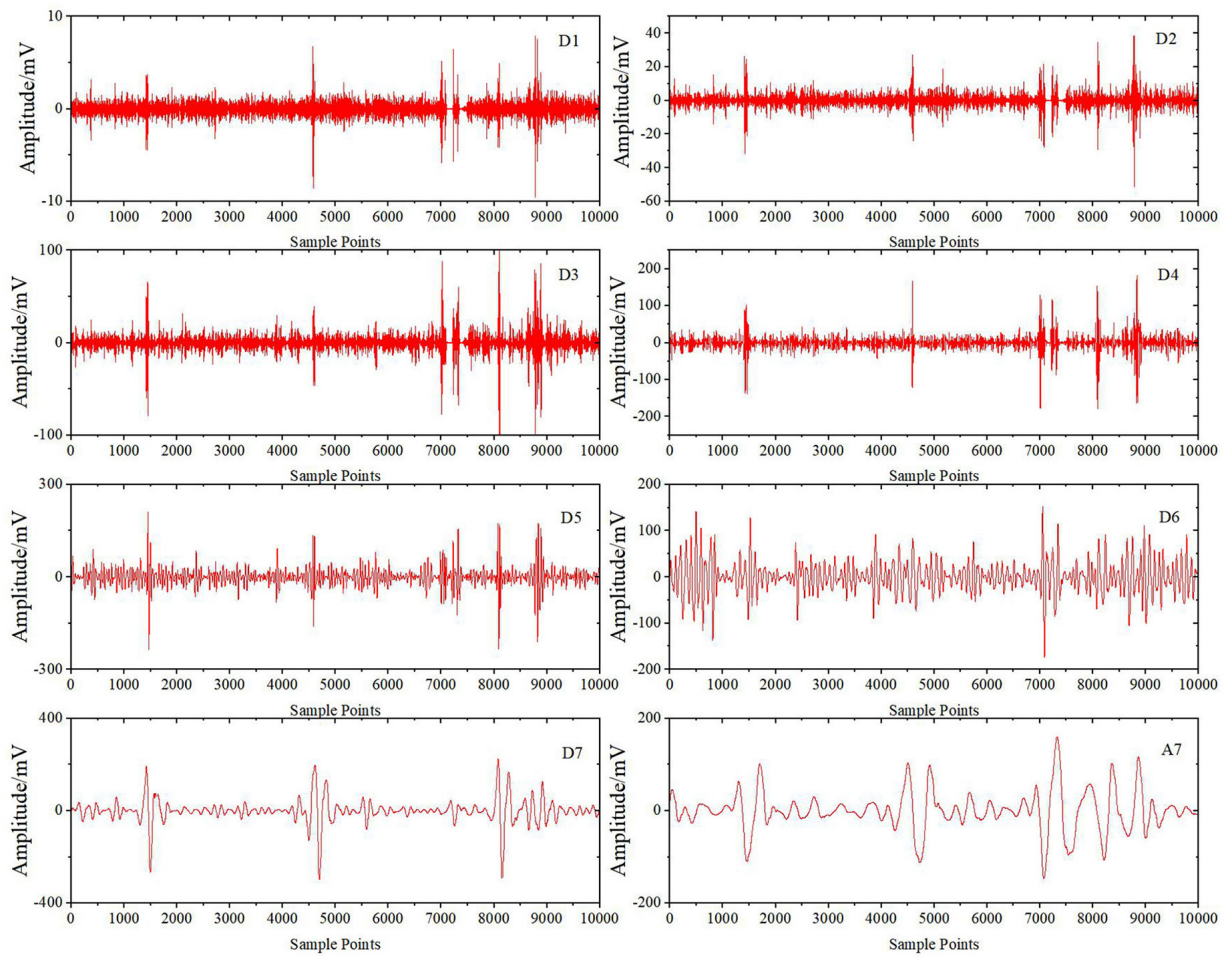
Wavelet decomposition results.

**Figure 7:**
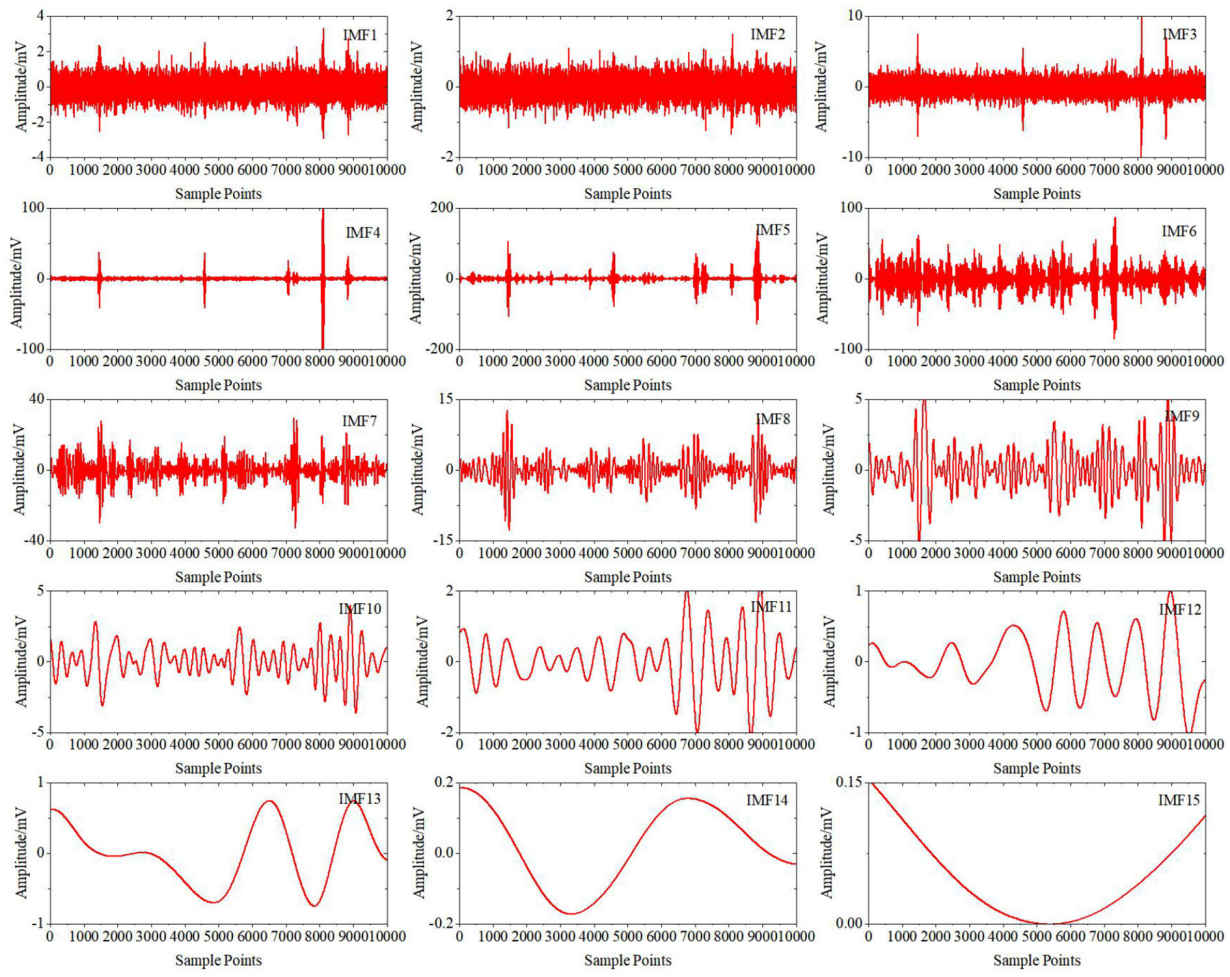
CEEMDAN decomposition results on high-frequency detail coefficient D5.

**Figure 8:**
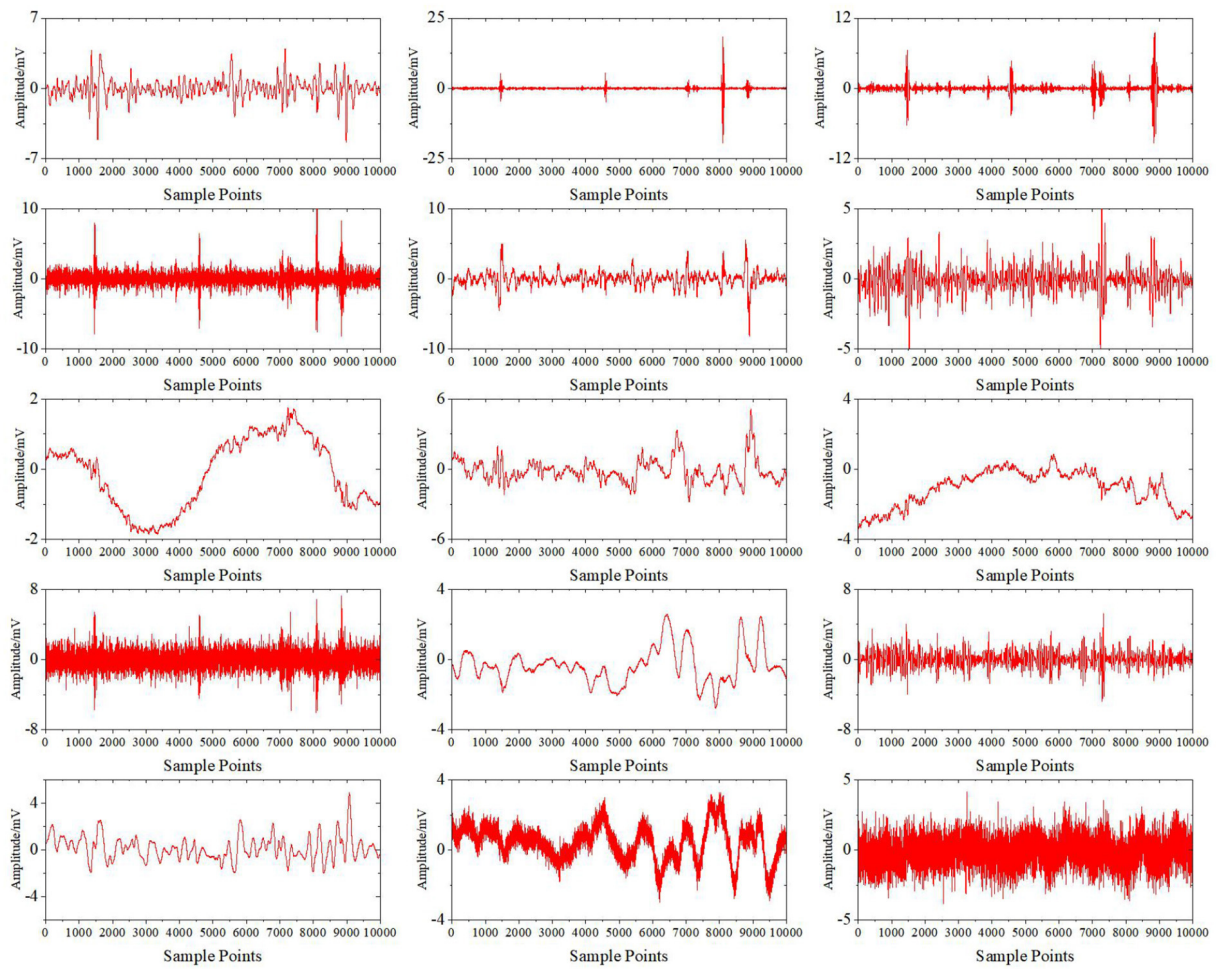
ICA decomposition results.

**Figure 9:**
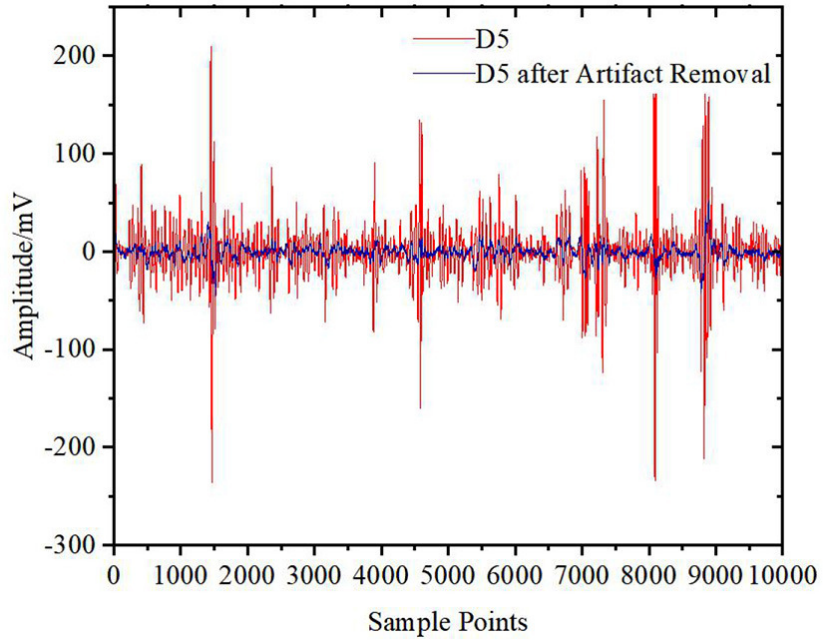
D5 signal and clean D5 signal.

Figure 6 shows the results of the preprocessed signals after wavelet decomposition. Then, CEEMDAN decomposition is carried out to obtain IMFi (i = 1.0.0.15) components for each wavelet coefficient. Take the high-frequency detail coefficient D5 as an example. Figure 7 shows the decomposition results. All the IMF components are decomposed by the ICA algorithm to obtain each independent component, as shown in Figure 8. Finally, the sample entropy of each ICA component is calculated, as shown in Table 1. From the table, we can see that the more complex the independent component is, the higher the sample entropy. Therefore, the component corresponding to the sample entropy satisfying the threshold discriminant is regarded as an EOG artifact and set to zero. Figure 9 shows the comparison result of D5 between its original value and the value after filtering the EOG artifacts by the DWT-CEEMDAN-ICA algorithm. We can see from the figure that our proposed algorithm has a very good effect in removing EOG artifacts.

**Table 1:** Sample entroy.

**Component**	**Entropy value**	**Component**	**Entropy value**	**Component**	**Entropy value**
ICA1	0.1226	ICA6	0.3407	ICA11	0.0429
ICA2	0.2962	ICA7	0.0218	ICA12	0.4822
ICA3	0.2241	ICA8	0.1047	ICA13	0.0567
ICA4	1.4709	ICA9	0.0633	ICA14	1.2973
ICA5	0.5457	ICA10	2.0395	ICA15	2.1393

**Figure 10:**
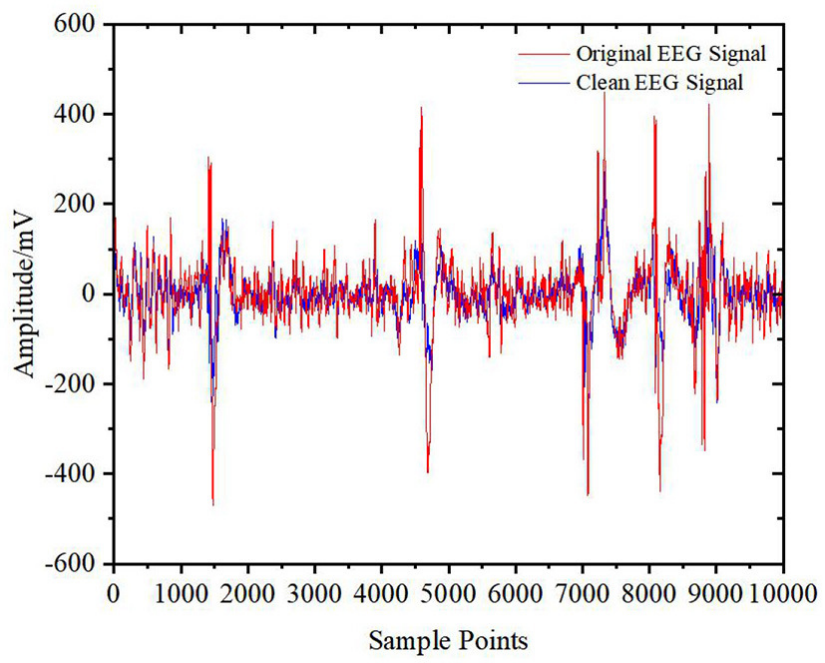
Original signal and clean signal.

However, there is still a gap between the original signals and the signals reconstructed only from the D5 coefficient. To prevent distortion and retain more effective EEG signals, other wavelet coefficients also need to be processed by the DWT-CEEMDAN-ICA algorithm and then reconstructed by wavelet reconstruction to obtain the final clean EEG signals without EOG artifacts. The result is shown in Figure 10. From this figure, it is shown that the algorithm removed EOG artifacts well and had a high degree of fitting for the EEG signals.

**Figure 11:**
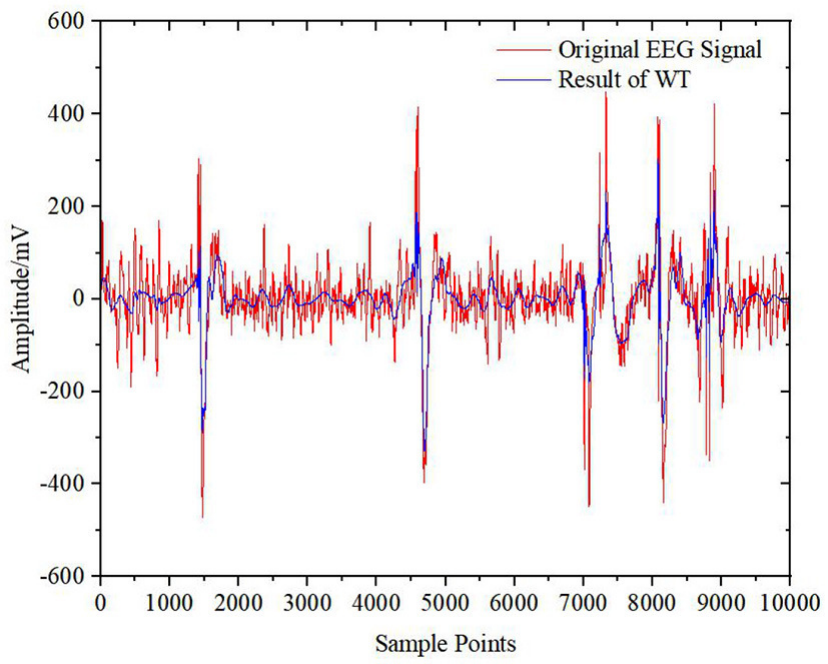
Raw EEG signal and result of WT.

**Figure 12:**
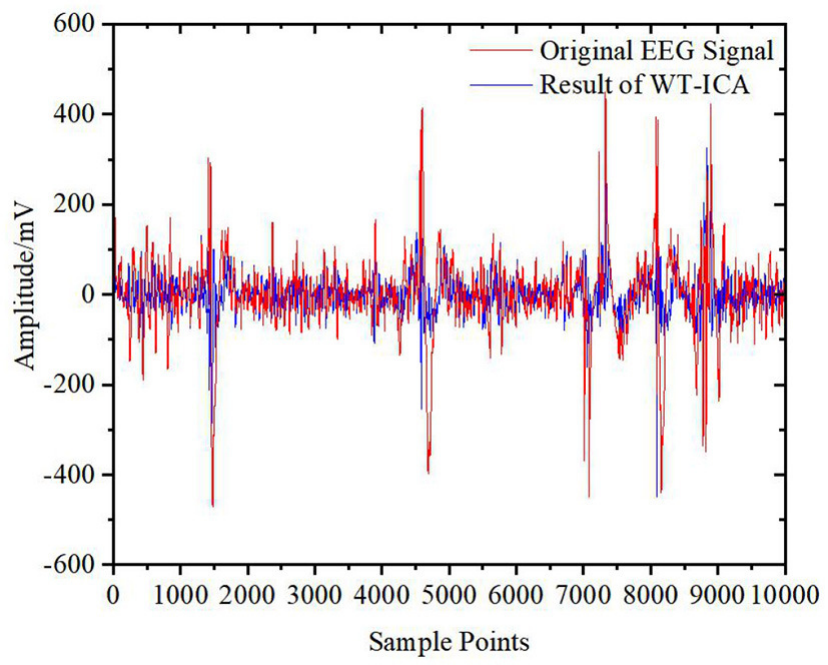
Raw EEG signal and result of WT-ICA.

**Figure 13:**
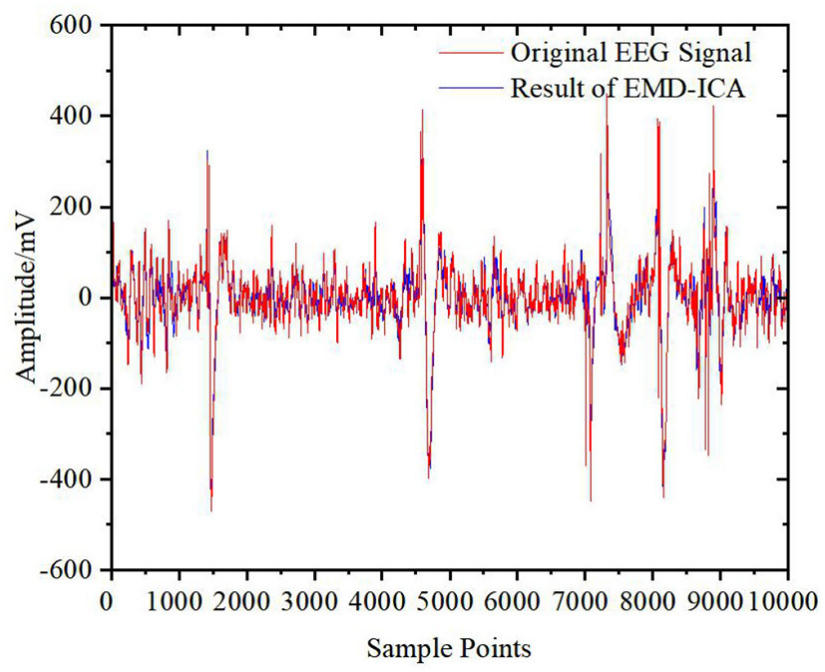
Original signal and EMD-ICA algorithm signal.

Figures 11–13 show the results of the other three algorithms on EOG artifact removal. We can see that the WT algorithm can not only effectively remove EOG artifacts but can also remove many valuable EEG signals, resulting in serious signal distortion. Moreover, this algorithm is highly subjective and the threshold and basis function need to be manually selected. Compared with the WT algorithm, the WT-ICA algorithm can retain more original EEG signals. Because of the problem of overcompleteness and the subjectivity of judgment, the effect is different every time, and the fitting of the original EEG signals is not good. The EMD-ICA algorithm cannot effectively remove EOG artifacts because of mode aliasing and noise.

**Figure 14:**
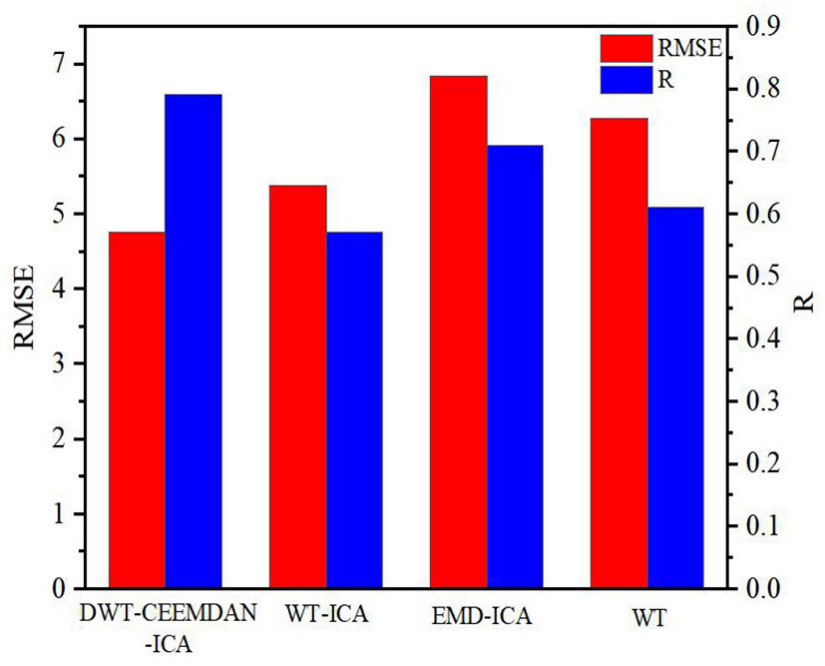
Comparison results between the errors and correlation coefficients of each algorithm.

Figure 14 shows the correlation coefficient R and root mean square error RMSE calculated by using the four algorithms for sampling points in the range of 50,000–55,000. The algorithm proposed in this paper obtains the largest correlation coefficient and the smallest root mean square error, which proves that it not only solves the overcomplete and modal aliasing problems but also effectively removes the EOG artifacts and retains more valuable original information.

## Conclusion

As a result of the problems of overcompleteness and mode aliasing in the single-channel EOG signal removal algorithm, the single-channel EEG equipment is restricted by few acquisition channels and lack of a reference electrode, so EOG artifacts cannot be effectively removed. We propose a novel method in this paper that integrates the discrete wavelet transform, complete empirical mode decomposition for adaptive noise, independent component analysis and the sample entropy algorithm. We carry out a series of experiments to demonstrate its effectiveness. Compared with some existing methods, our method can effectively identify and remove EOG artifacts from original signals while solving the above problems.

## Data availability statement

The raw data supporting the conclusions of this article will be made available by the authors, without undue reservation.

## Ethics statement

The studies involving human participants were reviewed and approved by School of Computer Science and Engineering, Guilin University of Aerospace Technology. The patients/participants provided their written informed consent to participate in this study.

## Author contributions

QH, ML, and YL: contributed to the design of this work. QH: methodology, validation, formal analysis, and writing—original draft. ML: software, data curation, and data analysis. YL: conceptualization, resources, writing—review and editing, and funding acquisition. All authors contributed to the article and approved the submitted version.

## Funding

This work was supported by the National Natural Science Foundation of China (81960324), General Project of Guangxi Natural Science Foundation (Guangdong-Guangxi Joint Fund, 2021GXNSFAA075037), and Guangxi University Middle-aged and Young Teachers' Basic Scientific Research Ability Improvement Project (2019KY0798, 2022KY0791, 2020KY21023, and 2019KY0822).

## Conflict of interest

The authors declare that the research was conducted in the absence of any commercial or financial relationships that could be construed as a potential conflict of interest.

## Publisher's note

All claims expressed in this article are solely those of the authors and do not necessarily represent those of their affiliated organizations, or those of the publisher, the editors and the reviewers. Any product that may be evaluated in this article, or claim that may be made by its manufacturer, is not guaranteed or endorsed by the publisher.
